# Exploration of the Use of Traditional Herbs to Overcome Cough and Cold in Three Provinces of East Java Province

**DOI:** 10.1155/tswj/1622754

**Published:** 2025-04-27

**Authors:** Wiwied Ekasari, Retno Widyowati, Nazila D. Kurnia, Akhmad Q. Jayanegara, Atikah L. Sari, Ram K. Sahu

**Affiliations:** ^1^Department of Pharmaceutical Sciences, Faculty of Pharmacy, Universitas Airlangga, Surabaya, East Java, Indonesia; ^2^Center of Natural Product Medicine Research and Development, Institute of Tropical Diseases, Universitas Airlangga, Surabaya, East Java, Indonesia; ^3^Department of Pharmaceutical Sciences, Hemvati Nandan Bahuguna Garhwal University (A Central University), Chauras Campus, Srinagar, Uttarakhand, India

## Abstract

This study is aimed at documenting traditional practices in the use of medicinal plants to overcome cough and cold in three selected regions of East Java Province, by establishing the relative significance, consensus, and scope of all medicinal plants used. The survey on the use of medicinal plants was conducted in one subdistrict in each of the three selected regions in East Java Province, Indonesia. Ethnomedicine data were collected through semistructured interviews, group discussions, and guided field visits from 111 informants. Plant importance is calculated using indices such as use report (UR), family importance value (FIV), and use value (UV). A total of 32 traditional herbs for the treatment of coughs and 20 traditional herbs for the treatment of colds, made of 25 species belonging to 21 genera from 15 families, have been identified as having ethnomedicine significance. The highest FIV (63.96) in the treatment of coughs and colds was reported for Zingiberaceae. The most commonly cited types of medicinal plants are *Kaempferia galanga* (27 UR; 0.27 UV) for the treatment of coughs and *Zingiber officinale* (34 UR; 0.486 UV) for the treatment of colds. The findings of this study show the rich tradition of using medicinal plants and cultural knowledge of local communities in three selected regions of East Java Province. Thus, for the potential management and conservation strategy of plant genetic resources, recording traditional knowledge about medicinal plants and their practices is very important. This legacy of awareness about medicinal plants will pave the way for future drug discovery to improve global health care.

## 1. Introduction

Cough is one of the most common medical complaints accounting for 30 million clinical visits per year. Cough is associated with various clinical associations and aetiologies. The aetiology of cough is largely based on the duration of cough, including acute cough if it occurs less than 3 weeks, subacute cough if it occurs for 3–8 weeks, and chronic cough if it occurs more than 8 weeks [1]. The most common cause of acute cough in adults is upper respiratory tract infections such as colds and acute bronchitis [2]. Colds are one of the viral infections that generally attack the upper respiratory tract. Adults catch colds about two to three times a year, whereas paediatrics can have 7–10 cases each year [3]. More than 200 viruses can cause the common cold in adults, including rhinoviruses (the most common cause), coronaviruses, adenoviruses, syncytial respiratory viruses, and parainfluenza viruses [4]. A cold is also defined as a short self-limiting and self-diagnosed mild illness with early symptoms of headache, sneezing, chills, and sore throat, followed by symptoms of nasal discharge, nasal congestion, cough, and malaise [5]. Generally, the severity of symptoms increases rapidly, peaking 2–3 days after infection, with an average duration of symptoms of 7–10 days, but with some symptoms persisting for more than 3 weeks [6].

Over the past 3 years to in the coming years, cough-cold and influenza will overlap with COVID-19, and both viruses present a global health threat, associated with a high risk of severe complications; both viruses are transmitted primarily through coughing, sneezing, and close body contact [7]. Based on a systematic literature review of the current evidence on the clinical presentation of COVID-19, the three main symptoms in patients with COVID-19 are fever (85.6%), cough (68.7%), and fatigue (39.4%) [8], followed by upper airway symptoms such as nasal congestion (3.7%).

Most cases of coughs and colds should be treated empirically and focus on reducing symptoms. For prevention, it can be done through physical interventions (for example, hand washing) and the possible use of zinc and honey supplements. For pharmacological treatment, acetaminophen and nonsteroidal anti-inflammatory drugs are generally used to treat pain and fever; dextromethorphan and guaifenesin as cough suppressants and expectorants; and the possibility of a combination of antihistamine-decongestants and intranasal ipratropium [9]. Currently, complementary medicine, including herbal medicine, is becoming an increasingly popular health care approach, which has been used both for general health maintenance and for the treatment of mild diseases such as coughs and colds [10]. Indonesia is known to have long used traditional medicine to treat various diseases. One of its provinces, East Java, was chosen as the study area because, according to statistical data from the Ministry of Home Affairs of the Republic of Indonesia in 2023 [11], the population of this region ranks second largest in Indonesia. Additionally, East Java ranks fourth among the provinces with the highest number of confirmed positive COVID-19 cases in Indonesia [12]. Currently, there are more than 250 traditional medicine producers in East Java Province, ranging from small-scale home industries to large-scale companies [13]. Furthermore, 79.8% of the population in East Java consumes *jamu* [14], including in areas such as Krian District in Sidoarjo Regency, Blimbing District in Malang City, and Tempurejo District in Jember Regency.

Data on the spread of COVID-19 shows that Sidoarjo Regency recorded 27,832 positive cases, with 1442 of those cases located in Krian District [15]. The use of medicinal plants in Krian District continues to be practiced, particularly during the COVID-19 pandemic in recent years. This was evident from a study conducted in Jatikalang Village, where a community service program promoted efforts to maintain immune health in order to prevent infection and alleviate mild symptoms such as cough and cold associated with COVID-19. These efforts included the consumption of *Tanaman Obat Keluarga* (TOGA), or Family Medicinal Plants, processed into *jamu beras kencur* (a traditional herbal drink made from rice and aromatic ginger) [16].

Blimbing District is located in the northern part of Malang City and is one of the three oldest districts in the city, serving as the gateway to Malang from the north. Blimbing District has the highest number of confirmed positive COVID-19 cases among the six districts in Malang City, with a total of 4547 cases and 257 reported deaths [17].

Meanwhile, Tempurejo District in Jember Regency has the largest area compared to the other study sites [18]. In a specific area of Andongrejo Village, there is a region known as the “Herbal Village” (*Kampung Herbal*) because the local community is accustomed to consuming and processing herbal plants for health purposes. According to data on the spread of COVID-19, Tempurejo District is among the 15 districts with the lowest number of cases in Jember Regency. Based on the tendency of people's back-to-nature lifestyles by utilizing various existing medicinal plants and cases of COVID-19, the researchers made three regions in East Java Province the object of research to find traditional herbs used by the community when experiencing cough-cold disease. The research was conducted as an effort to identify single plants and traditional medicinal herbs used in overcoming coughs and colds before and during the COVID-19 pandemic. The approach is done by identifying the use of medicinal plants commonly used by the community to overcome coughs and colds. The research will be conducted by interviewing informants to obtain data on medicinal plants related to the type of plant used, the purpose of use, the part of the plant used, and the method of manufacture and use.

## 2. Materials and Methods

### 2.1. Study Area

Fieldwork was carried out in East Java Province, Indonesia (7°12⁣′–8°48⁣′ S 111°0⁣′–114°4⁣′ E), bordering the Java Sea to the north, the Indian Ocean to the south, Central Java Province to the west, and the Bali Strait to the east [19]. This province has two main regions, namely, mainland East Java and the Madura Islands. East Java Province is divided into 29 regencies and 9 cities ranging from the largest to the smallest land area: Banyuwangi, Malang, Jember, Bojonegoro, Sumenep, Tuban, Lumajang, Lamongan, Probolinggo, Situbondo, Bondowoso, Pasuruan, Pacitan, Kediri, Blitar, Ponorogo, Ngawi, Sampang, Nganjuk, Gresik, Trenggalek, Jombang, Tulungagung, Madiun, Bangkalan, Pamekasan, Mojokerto, Magetan, and Sidoarjo; followed by Surabaya, Malang, Batu, Kediri, Probolinggo, Pasuruan, Madiun, Blitar, and Mojokerto.

**Figure 1:**
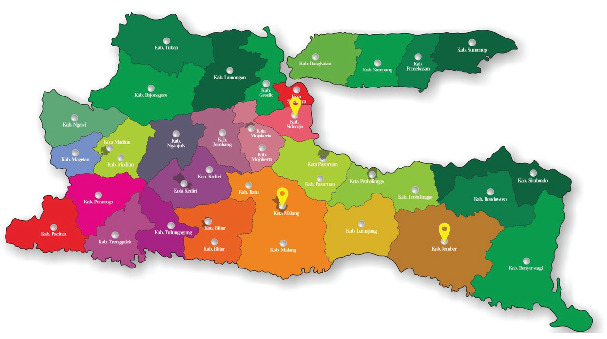
Selected sites of East Java Province: Sidoarjo Regency, Malang City, and Jember Regency.

This study deliberately included each of the selected districts in three selected regencies/cities, namely, Krian District in Sidoarjo Regency, Blimbing District in Malang City, and Tempurejo District in Jember Regency (see Figure 1).

Krian District, located in Sidoarjo Regency, lies between 112°35⁣′44⁣^″^ E longitude and 7°23⁣′53⁣^″^ S latitude, covering an area of 32.50 km^2^ with a population of 130,412 according to the 2023 national census [20]. Geographically, Krian District occupies a strategic location as it sits between four major regency/city capitals: Surabaya (to the east), Sidoarjo (to the south), Gresik (to the north), and Mojokerto (to the west). Its strategic position also makes it an important transportation hub, as it lies along one of the main transportation routes on the southern corridor of Java Island. Due to this advantageous location, Krian District serves as the economic center for communities in western Sidoarjo and plays a key role in the economic activities of neighboring areas.

Blimbing District, in Malang City, lies between 112°39⁣′1⁣^″^ E longitude and 7°56⁣′50⁣^″^ S latitude. It covers an area of 17.76 km^2^ with a population of 190,799 according to the 2024 national census [18]. Blimbing District is located in the northern part of Malang City and is one of the three oldest districts in the area, having been established when Malang was designated as a municipality. Its strategic location makes Blimbing District the northern gateway into Malang City. Meanwhile, Tempurejo District, in Jember Regency, is situated in the southern part of Jember. It lies between 113°42⁣′43⁣^″^ E longitude and 8°20⁣′17⁣^″^ S latitude, with an elevation ranging from approximately 25–1000 m above sea level [21]. In all three areas, the majority of the population relies on agriculture or farming as their primary source of livelihood. The study areas experience two distinct tropical seasons: the rainy season (November to April) and the dry season (May to October). Air temperatures across these regions typically range from 21°C to 34°C, accompanied by high humidity. The Javanese ethnic group is the predominant population in all three study areas. As a result, Javanese is one of the two main languages spoken in East Java and is the dominant language in these regions. The majority of residents in these districts adhere to Islam, Christianity, or Catholicism, while a smaller percentage practice Hinduism or Buddhism. Despite having access to healthcare facilities such as hospitals and community health centers (*puskesmas*), a significant portion of the population in these areas still relies heavily on traditional practitioners for their healthcare needs, largely due to limited access to modern healthcare services.

### 2.2. Survey Design

This research method is an exploratory survey with data collection techniques using interviews, observations, or direct observations in the field, as well as literature studies. Prior to actual interviews, field surveys, and selection in selected areas in East Java Province, the research protocol was approved by the Health Research Ethics Commission of the Faculty of Pharmacy, Universitas Airlangga, and permits from each regional leader have been obtained. Meetings and deliberations were held together with one regional leader and key informants in each region.

Fieldwork during March to August 2023 has been carried out. Informants were determined by the purposive sampling method using the snowball technique [22] based on information from the linker. Informants were selected based on experience in the use of traditional medicine with inclusion criteria, namely, local people who use traditional herbs to overcome colds for more than 3 years, willing to be involved in this study, men or women, and adults (≥ 18 years old) or married. Semistructured interviews use instruments in the form of questionnaires with semiopen questions related to demographic data and local knowledge information about medicinal plants or traditional herbs used to treat cough and cold (see Supporting Information (available here)).

Direct observation or observation in the field by collecting specimens (medicinal plants or as traditional herbs) and documenting them for further identification refers to various relevant literature sources. These plants were identified using standard flora available in the Indonesian region, namely, Flora of Java [23]. Furthermore, the taxonomic name of the plant species was confirmed in the pharmacognosy and phytochemistry laboratory, Faculty of Pharmacy, Universitas Airlangga, Indonesia, from the newly created online database World Flora Online [24] (http://www.worldfloraonline.org/) as a replacement for The Plant List (http://www.plantlist.org/). All preserved specimens were deposited in the Pharmacognosy and Phytochemistry Laboratory of the Faculty of Pharmacy, Universitas Airlangga.

### 2.3. Statistical Analysis

The results and discussion may be presented separately, or in one combined section, and may optionally be divided into headed subsections.

Indigenous knowledge is assessed quantitatively using various indices such as use report (UR), family importance value (FIV), and use value (UV). UR was calculated to determine the specific frequency of use of each species reported by informants [25]. FIV is calculated to identify the most important families according to the number of informant citation reports [26]. This value is determined using Equation (1).

(1) $$\begin{array}{c} \text{FIV}=\frac{{\text{FC}}_{\text{family}}}{N}\times 100 \end{array}$$

where FC_family_ is the number of informants who mention the family, while *N* is the total number of informants who participated in the study.

UV was calculated to determine the relative importance of locally known species [27], based on Equation (2).

(2) $$\begin{array}{c} \text{UV}=\frac{U_{i}}{N} \end{array}$$

where *U*_*i*_ is the number of usage reports mentioned by each informant, while *N* is the total number of informants participating in the study. UV is generally high, that is, close to 1 if the amount of use is high and close to 0 if the report of use for a species is very low.

## 3. Results and Discussion

The preservation of ethnomedicinal knowledge in Indonesia largely depends on the region [28]. In some rural areas, traditional knowledge remains well-preserved, while in urban areas, it is gradually declining. This trend is not uniform across the country, as some regions have taken more active steps to preserve their cultural heritage [29–32] compared to others.

### 3.1. Sociodemographic Profile of Informants

**Figure 2:**
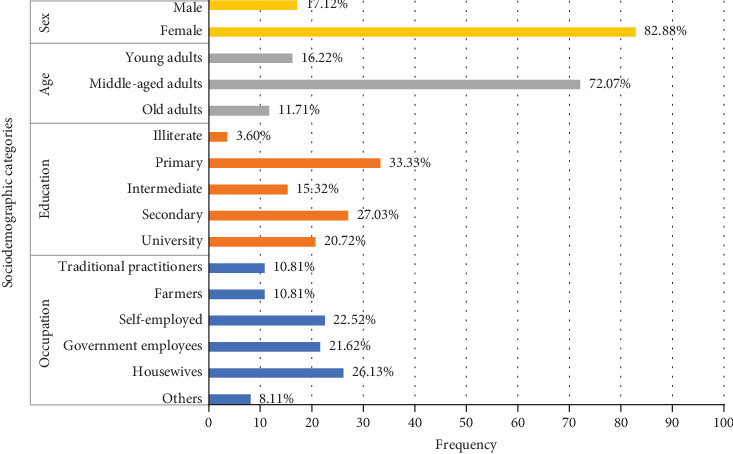
Sociodemographic profile of informants (*n* = 111) in three selected regions of East Java Province.

A total of 111 informants were interviewed and categorized into different demographic categories (see Figure 2). There were 17.12% male informants and 82.88% female informants. Based on age, informants were grouped into three large groups, namely, young adults aged 17–30 years (16.22%), middle-aged adults aged 31–59 years (72.07%), and older adults aged 60 years and over (11.71%).

Informants also have different educational backgrounds. Local wisdom about the use of medicinal plants for the treatment of cough and cold is more prevalent in people with elementary or primary education level (33.33%), and the same knowledge decreased in the higher education group in the area, with junior high school or intermediate level (15.32%) followed by high school or secondary level (27.03%) and university level (20.72%). The number of people who did not take formal education was 3.60%. Housewives (26.13%) represented the most informant professions, followed by self-employed (22.52%), government employees (21.62%), traditional practitioners and farmers (10.81%), and other professions (8.11%).

### 3.2. Medicinal Plant Documentation

The documentation of medicinal knowledge regarding the use of native plants for treating various diseases by local communities within a specific region or culture—referred to as ethnomedicine—can serve as one of the primary means to improve public health [33]. Often, due to concerns over losing clientele and the risk of exposing their sources of income or livelihood, individuals who possess medicinal knowledge about the use of native plants consider this knowledge a family secret that should not be disclosed, but rather protected and passed down to family members. In some cases, this knowledge is transmitted orally without proper documentation. However, ethnomedicinal surveys have played a critical role in preventing the loss of indigenous knowledge. This study documents the medicinal plants used in the management of coughs and colds, as well as the existing knowledge among communities in three selected regions of East Java Province. Based on information from respondents, there were 32 traditional herbs to overcome cough in three selected regions in East Java Province (see Table 1). The most popular traditional concoction mentioned by 18 informants is a simple concoction made from one tablespoon of *Citrus aurantiifolia* fruit juice mixed with sweet soy sauce to taste. This herb can be taken three times a day when cough complaints appear.

Based on information from informants, there were 20 traditional herbs to overcome colds in three selected regions in East Java Province (see Table 2). In contrast to cough treatments that have the same ingredients in each region, traditional herbs for cold treatment are reported to have the most popular ingredients that differ from the study area. In Krian District, Sidoarjo Regency, there are only two herbs to overcome colds. Both contain the same ingredients, namely, fresh rhizomes from *Curcuma zanthorrhiza* and *Curcuma longa*, *Cymbopogon citratus* herbs, and *Tamarindus indica* fruit processed in the same way. The only thing that distinguishes is the addition of *Citrus aurantiifolia* fruit in the first recipe, RPS01.

Meanwhile, in the Blimbing District, Malang City, there are the most popular traditional ingredients mentioned by three out of 31 respondents, containing *Zingiber officinale* rhizomes, *Cymbopogon citratus* herbs, and *Cinnamomum burmanni* bark, which are boiled together. This herb can be consumed twice a day as long as complaints of colds appear. As well as in the Tempurejo District, Jember Regency, there are the most popular traditional ingredients mentioned by three out of 35 respondents, which are simple ingredients made from *Citrus aurantiifolia* fruit juice mixed with sweet soy sauce to taste. This herb can be drunk when complaints of colds appear.

### 3.3. Diversity of Medicinal Plant Species to Treat Coughs and Colds in Three Selected Regions of East Java Province

**Figure 3:**
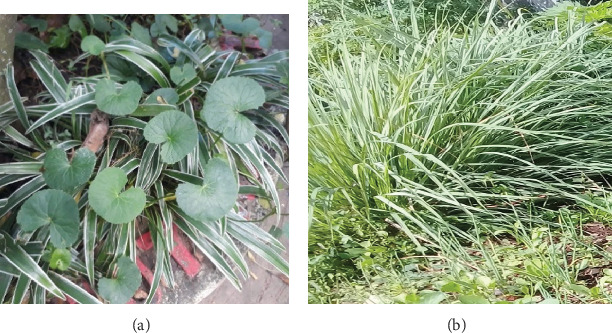
Several medicinal plant species used in the treatment of coughs and colds in three selected regions of East Java Province: (a) *Centella asiatica*; (b) *Cymbopogon citratus*.

Overall, this study reported the utilization of 25 species of medicinal plants distributed in 21 genera included in 15 families for the treatment of coughs and colds in three regions of East Java Province (see Table 3 and Figure 3). There were 27 reports of the use of *Kaempferia galanga* for the treatment of cough and 34 reports of the use of *Zingiber officinale* for the treatment of colds. It was also observed that several species were used to treat both ailments, namely, coughs and colds, including *Cinnamomum burmanni*, *Tamarindus indica*, *Allium sativum*, *Cymbopogon citratus*, *Nigella sativa*, *Citrus aurantiifolia*, *Amomum compactum*, *Boesenbergia pandurata*, *Curcuma longa*, *Curcuma zanthorrhiza*, *Kaempferia galanga*, and *Zingiber officinale*.

Several plants mentioned in this study have also been cited in similar works [34–36], supporting the traditional use of four species among the 25 identified in this study for managing coughs and colds in India. These species include *Allium sativum*, *Curcuma longa*, *Piper nigrum*, and *Zingiber officinale*. In addition, in traditional Brazilian medicine, *Zingiber officinale* has been used to manage coughs and colds in children [37]. Therefore, the use of the same plant species across different cultures in the management of coughs and colds strongly suggests their potential effectiveness.

Reviews on the plants mentioned in this study indicate that *Elephantopus scaber* [38], *Cinnamomum burmanni* [39], *Averrhoa bilimbi* [40], *Peperomia pellucida* [41], *Cymbopogon citratus* [42], *Morinda citrifolia* [43, 44], *Citrus aurantiifolia* [45], *Curcuma zanthorrhiza* [46], *Curcuma zedoaria* [47], *Kaempferia galanga* [48–50], *Zingiber officinale* [51, 52], and *Zingiber officinale* var. *rubrum* [53] possess antitussive and anticold properties. Other plant species mentioned in this study have also been investigated scientifically in both preclinical [54–97] and clinical studies [98–102] by previous researchers and have been reported to exhibit antitussive and anticold properties. These findings cover 96% of the total plant species involved in this study, and active compounds have also been identified from several of these plants. For instance, arabinogalactan Type II pectic polysaccharides from *Piper nigrum* [63], thymoquinone from *Nigella sativa* [67–69], flavonoids from *Citrus aurantiifolia* [72], capsaicinoids from *Capsicum frutescens* [74], and curcumin from *Curcuma longa* [81] are among the phytochemicals that have demonstrated antitussive and anticold activities.

The effectiveness of the remaining 4% of plant species still requires further scientific investigation to confirm their potential in the management of coughs and colds.

### 3.4. Parts Used, Method of Preparation, and Route of Administration

**Figure 4:**
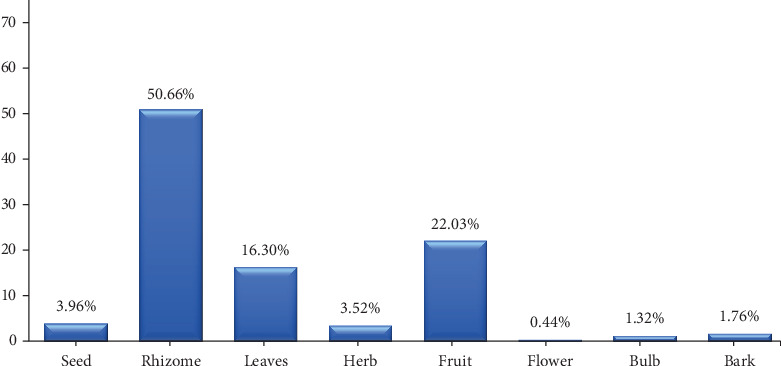
Plant parts used in the treatment of coughs and colds in three selected regions of East Java Province.

Among the various plant parts used, rhizomes (50.66%) are most often used for making traditional herbs singly or mixed with other plant parts (see Figure 4). Other plant parts, such as fruit (22.03%) followed by leaves (16.30%), seeds (3.96%), herbs (3.52%), stem bark (1.76%), bulbs (1.32%), and flowers (0.44%), were also reported for use by respondents in the study area. A similar study conducted in Darjeeling, in the eastern Himalayas of India [35], also reported this observation. Moktan and Rai [35] noted that in traditional medicine practices for treating respiratory diseases and disorders, including coughs and colds, the most commonly used plant parts are rhizomes and roots. This may be attributed to their ease of harvesting; they are readily accessible underground and can easily regenerate new plants, allowing for sustainable harvesting practices. Additionally, rhizomes are frequently used in traditional medicine because they often contain high concentrations of bioactive compounds and serve as nutrient storage organs in plants, making them valuable resources for medicinal purposes across various cultures [103].

**Figure 5:**
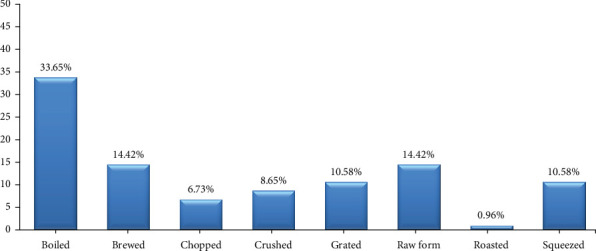
Methods of preparing medicinal plants reported to treat coughs and colds in three selected regions of East Java Province.

The most commonly used method of processing medicinal plant parts was boiled (33.65%) followed by brewed or unprocessed (14.42%), squeezed or shredded (10.58%), pounded (8.65%), chopped (6.73), and baked (0.96%) (see Figure 5). The time required for boiling typically varies depending on the plant materials or specific plant parts being processed.

**Figure 6:**
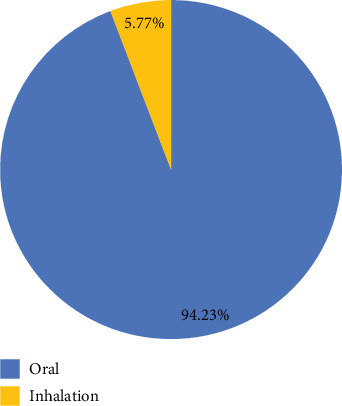
Route of administration reported to treat coughs and colds in three selected regions of East Java Province.

Internal use is more than external use. Most traditional herbs are given orally by drinking or eating directly (94.23%) (see Figure 6). A full cup of tea (200 mL), taken one to three times a day for a duration based on individual needs, was the dosage recommended by several respondents. Local communities and traditional health practitioners often use several auxiliary ingredients such as honey, sugar, salt, and eucalyptus oil to increase the adequacy and efficacy of certain therapeutic drugs. In addition, the use of inhalation (5.77%) is also done by brewing plant parts in hot water and inhaling the steam through the nose in the treatment of colds.

During the study, it was observed that the local community demonstrated an efficient approach to processing plants and their parts for use as herbal medicine. Their strong traditional beliefs in ethnobotanical practices appeared to be highly motivating. Therefore, raising awareness about cultivating more ethnobotanically significant plant species in their fields and gardens should be encouraged.

### 3.5. FIV

**Figure 7:**
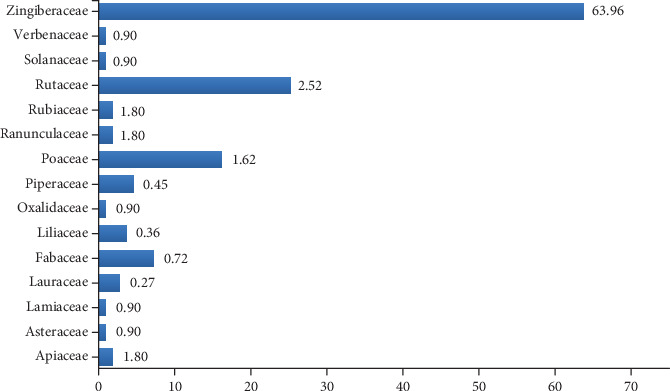
Family importance value (FIV).

The most common family as described by its FIV is Zingiberaceae as the dominant family with 63.96 FIV followed by Rutaceae (25.23), Poaceae (16.22), Fabaceae (7.2), Piperaceae (4.50), and Liliaceae (3.60) (see Figure 7). The smallest FIV values were observed in Asteraceae (0.90) followed by Lamiaceae (0.90), Oxalidaceae (0.90), Solanaceae (0.90), Verbenaceae (0.90), Apiaceae (1.80), Ranunculaceae (1.80), Rubiaceae (1.80), and Lauraceae (2.70). These families were consistently recognized by the informants for their effectiveness in treating coughs and colds. A similar ethnomedicinal study by Wahyuningrum et al. [104] reported the extensive use of Zingiberaceae family members for treating respiratory diseases—particularly coughs and colds—in Cilongok Subdistrict, Banyumas Regency, Central Java Province, Indonesia. The use of Zingiberaceae members in the treatment of coughs and colds has also been documented by other researchers in the upstream areas of the Bengawan Solo River, Central Java Province, Indonesia [105], as well as in Srikakulam District, Andhra Pradesh, India [34]. In addition, an ethnobotanical analysis documented by Jenipher and Ayyanar [106] on the treatment of respiratory diseases among the Kani tribal communities in Tirunelveli District (Tamil Nadu, India) identified Rutaceae and Fabaceae as dominant families, similar to the findings of this study. Scientific confirmation of several species within the same genera and families may indicate comparable therapeutic effects that can be utilized in the treatment of various diseases and health conditions [107].

Zingiberaceae was identified as the most widely used plant family by informants in the three selected areas of East Java Province, as species from this family are relatively easy to cultivate in the region. These plants are abundant throughout Indonesia due to the country's tropical climate, which is highly suitable for the growth of various Zingiberaceae species [108].

## 4. Conclusions

Traditional herbs to overcome cough in three regions of East Java Province recorded a total of 32 recipes, while traditional herbs to overcome colds recorded a total of 20 recipes. Of these traditional herbs, a total of 25 species of medicinal plants in 21 genera in 15 families are used as both single and mixed medicinal ingredients. The results showed that *Kaempferia galanga* was predominantly used to treat coughs, and *Zingiber officinale* was predominantly used to treat colds, where rhizomes were the most commonly used plant parts. The most common processing method is by boiling medicinal plant ingredients and is generally consumed orally. In general, no adverse effects were reported by the informant.

This is the first survey conducted in this region and the area as a whole, to the best of our knowledge. This study contributes valuable knowledge regarding the use of therapeutic plant species for the treatment of coughs and colds. The ethnorespiratory knowledge documented here aligns with several other ethnomedicinal surveys conducted in neighboring regions and around the world. However, this survey specifically reports a total of 25 therapeutic plant species traditionally used for managing coughs and colds. The data highlight the variability in the plant parts utilized, methods of preparation by indigenous people, and modes of administration across the region and globally, providing new ethnomedicinal insights.

## Figures and Tables

**Table 1:** The composition, processing, and use of traditional herbs to overcome cough reported by respondents in three selected regions in East Java Province.

**Traditional herbs code number**	**Composition and method of preparation**	**Dosage**	**No. of respondents who cited**
*Krian District, Sidoarjo Regency*			
RBS01	Fresh *Kaempferia galanga* rhizomes are pounded or crushed, then brewed with boiling water, and mixed with sugar to taste	Taken while coughing, one cup at a time	3
RBS02	Fresh *Zingiber officinale* rhizomes and *Cymbopogon citratus* herbs are crushed, then brewed with boiling water, and mixed with sugar to taste	Taken while coughing	1
RBS03	Fresh *Zingiber officinale* rhizomes are shredded, squeezed, and then mixed with a cold decoction of brown sugar	Taken when coughing, three times two tablespoons a day	1
RBS04	Fresh *Zingiber officinale* rhizomes are crushed, then brewed with boiling water, and mixed with sugar to taste	Taken while coughing	2
RBS05	One tablespoon of fresh *Citrus aurantiifolia* fruit juice is mixed with sweet soy sauce to taste	Taken while coughing	3
RBS06	Fresh rhizomes from *Zingiber officinale* and *Curcuma longa*, *Cymbopogon citratus* herbs, and *Tamarindus indica* fruit are cut into small pieces, boiled, then filtered, and mixed with sugar to taste	Taken while coughing, with or without vitamins	1
RBS07	*Jamu Beras Kencur*: *Oryza sativa* seeds, *Kaempferia galanga* rhizomes, *Zingiber officinale* rhizomes, *Allium sativum* bulbs, *Tamarindus indica* fruit, and *Piper nigrum* seeds are pounded, boiled, cooled, filtered, mixed with salt and sugar to taste, and then precipitated	Taken while coughing	1
RBS08	Fresh *Zingiber officinale* var. *rubrum* rhizomes are crushed, then brewed with boiling water, and mixed with sugar to taste	Taken while coughing	1
RBS09	*Jamu Beras Kencur*: *Oryza sativa* seeds, *Kaempferia galanga* rhizomes, *Zingiber officinale* rhizomes, *Allium sativum* var. Solo garlic bulbs, and *Tamarindus indica* fruit are pounded, boiled with brown sugar, cooled, filtered, and then mixed with *Citrus aurantiifolia* fruit juice, salt and sugar to taste	Taken while coughing	1
RBS10	*Jamu Sinom*: Fresh rhizomes from *Curcuma longa*, *Curcuma zanthorrhiza*, and *Curcuma zedoaria* are pounded with fresh young leaves from *Tamarindus indica* and *Elephantopus scaber*, boiled with water and ripe *Tamarindus indica* fruit, filtered, and then mixed with juice of *Citrus aurantiifolia* fruit, salt and sugar to taste	Taken while coughing	1
RBS11	*Citrus aurantiifolia* fruit is roasted, cut, and then squeezed directly into the mouth	Taken while coughing	1

*Blimbing District, Malang City*			
RBM01	Fresh *Curcuma longa* rhizomes are shredded, mixed with a little boiled water, squeezed, and then mixed with honey to taste	Taken while coughing, two times a day after meals	1
RBM02	Fresh *Zingiber officinale* rhizomes are chopped, boiled with *Cymbopogon citratus* herbs, and then mixed with *Citrus aurantiifolia* fruit juice	Taken once a week	1
RBM03	*Jamu Beras Kencur* (main ingredient of *Oryza sativa* seeds and *Kaempferia galanga* rhizome) mixed with *Cymbopogon citratus* leaf decoction	Taken two times a day	1
RBM04	*Citrus aurantiifolia* fruit juices are mixed with sweet soy sauce to taste	Taken while coughing, three times a day	1
RBM05	Fresh rhizomes from *Zingiber officinale*, *Kaempferia galanga*, *Curcuma zanthorrhiza*, *Curcuma longa*, and *Boesenbergia pandurata* are pounded with *Tamarindus indica* fruit and then brewed with hot water	Taken two times a day	1
RBM06	Fresh *Zingiber officinale* rhizomes are chopped, mixed with *Piper nigrum* seed powder and *Citrus aurantiifolia* fruit juice, boiled, and then mixed with honey to taste	Taken three times a day	2
RBM07	Fresh leaves of *Stachytarpheta jamaicensis* are cut into small pieces, brewed, and then mixed with *Citrus aurantiifolia* fruit juice	Taken two times a day	1
RBM08	Fresh *Curcuma longa* rhizomes are chopped and then boiled with fresh *Phyllanthus niruri* herbs	Taken two times a day	1
RBM09	Fresh *Kaempferia galanga* rhizomes are sautéed and then boiled with small pieces of *Tamarindus indica* fruit	Taken more than three times a day	1
RBM10	Fresh *Zingiber officinale* rhizomes are shredded, mixed with *Citrus aurantiifolia* fruit juice, and then brewed with hot water	Taken two times a day	1
RBM11	Fresh *Zingiber officinale* rhizomes are chopped and then boiled with *Cymbopogon citratus* herbs and *Cinnamomum burmanni* bark	Taken two times a day	3

*Tempurejo District, Jember Regency*			
RBJ01	Fresh rhizomes from *Zingiber officinale* and *Curcuma longa* are boiled or brewed together	Taken while coughing	1
RBJ02	*Citrus aurantiifolia* fruit juices are mixed with sweet soy sauce to taste	Taken while coughing	14
RBJ03	Fresh rhizomes from *Curcuma longa* and *Kaempferia galanga* are shredded and then squeezed	Taken while coughing	1
RBJ04	Fresh *Kaempferia galanga* rhizomes are shredded, then squeezed, and mixed with honey to taste	Taken while coughing	2
RBJ05	Fresh *Zingiber officinale* rhizomes are thinly sliced, then brewed, and mixed with honey to taste	Taken while coughing	1
RBJ06	Fresh *Zingiber officinale* var. *rubrum* rhizomes are boiled with *Cymbopogon citratus* herbs	Taken while coughing	1
RBJ07	Fresh *Peperomia pellucida* herbs are shredded with fresh *Kaempferia galanga* rhizomes and then squeezed	Taken while coughing	1
RBJ08	Fresh *Peperomia pellucida* herbs are boiled with fresh *Zingiber officinale* rhizomes	Taken while coughing	1
RBJ09	*Oryza sativa* seeds are soaked with water, drained, pounded with fresh *Kaempferia galanga* rhizomes, and then squeezed	Taken while coughing	1
RBJ10	Fresh rhizomes from *Kaempferia galanga*, *Curcuma zanthorrhiza*, and *Curcuma longa* are boiled with *Amomum compactum* fruit	Taken while coughing	1

**Table 2:** The composition, processing, and use of traditional herbs to overcome colds reported by respondents in three selected regions in East Java Province.

**Traditional herbs code number**	**Composition and method of preparation**	**Dosage**	**No. of respondents who cited**
*Krian District, Sidoarjo Regency*			
RBS01	Fresh *Kaempferia galanga* rhizomes are pounded or crushed, then brewed with boiling water and mixed with sugar to taste	Taken while coughing, one cup at a time	3
RBS02	Fresh *Zingiber officinale* rhizomes and *Cymbopogon citratus* herbs are crushed, then brewed with boiling water, and mixed with sugar to taste	Taken while coughing	1
RBS03	Fresh *Zingiber officinale* rhizomes are shredded, squeezed, and then mixed with a cold decoction of brown sugar	Taken when coughing, three times two tablespoons a day	1
RBS04	Fresh *Zingiber officinale* rhizomes are crushed, then brewed with boiling water, and mixed with sugar to taste	Taken while coughing	2
RBS05	One tablespoon of fresh *Citrus aurantiifolia* fruit juice is mixed with sweet soy sauce to taste	Taken while coughing	3
RBS06	Fresh rhizomes from *Zingiber officinale* and *Curcuma longa*, *Cymbopogon citratus* herbs, and *Tamarindus indica* fruit are cut into small pieces, boiled, then filtered, and mixed with sugar to taste	Taken while coughing, with or without vitamins	1
RBS07	*Jamu Beras Kencur*: *Oryza sativa* seeds, *Kaempferia galanga* rhizomes, *Zingiber officinale* rhizomes, *Allium sativum* bulbs, *Tamarindus indica* fruit, and *Piper nigrum* seeds are pounded, boiled, cooled, filtered, mixed with salt and sugar to taste, and then precipitated	Taken while coughing	1
RBS08	Fresh *Zingiber officinale* var. *rubrum* rhizomes are crushed, then brewed with boiling water, and mixed with sugar to taste	Taken while coughing	1
RBS09	*Jamu Beras Kencur*: *Oryza sativa* seeds, *Kaempferia galanga* rhizomes, *Zingiber officinale* rhizomes, *Allium sativum* var. Solo garlic bulbs, and *Tamarindus indica* fruit are pounded, boiled with brown sugar, cooled, filtered, and then mixed with *Citrus aurantiifolia* fruit juice, salt and sugar to taste	Taken while coughing	1
RBS10	*Jamu Sinom*: Fresh rhizomes from *Curcuma longa*, *Curcuma zanthorrhiza*, and *Curcuma zedoaria* are pounded with fresh young leaves from *Tamarindus indica* and *Elephantopus scaber*, boiled with water and ripe *Tamarindus indica* fruit, filtered, and then mixed with juice of *Citrus aurantiifolia* fruit, salt and sugar to taste	Taken while coughing	1
RBS11	*Citrus aurantiifolia* fruit is roasted, cut, and then squeezed directly into the mouth	Taken while coughing	1

*Blimbing District, Malang City*			
RBM01	Fresh *Curcuma longa* rhizomes are shredded, mixed with a little boiled water, squeezed, and then mixed with honey to taste	Taken while coughing, two times a day after meals	1
RBM02	Fresh *Zingiber officinale* rhizomes are chopped, boiled with *Cymbopogon citratus* herbs, and then mixed with *Citrus aurantiifolia* fruit juice	Taken once a week	1
RBM03	*Jamu Beras Kencur* (main ingredient of *Oryza sativa* seeds and *Kaempferia galanga* rhizome) mixed with *Cymbopogon citratus* leaf decoction	Taken two times a day	1
RBM04	*Citrus aurantiifolia* fruit juices are mixed with sweet soy sauce to taste	Taken while coughing, three times a day	1
RBM05	Fresh rhizomes from *Zingiber officinale*, *Kaempferia galanga*, *Curcuma zanthorrhiza*, *Curcuma longa*, and *Boesenbergia pandurata* are pounded with *Tamarindus indica* fruit and then brewed with hot water	Taken two times a day	1
RBM06	Fresh *Zingiber officinale* rhizomes are chopped, mixed with *Piper nigrum* seed powder and *Citrus aurantiifolia* fruit juice, boiled, and then mixed with honey to taste	Taken three times a day	2
RBM07	Fresh leaves of *Stachytarpheta jamaicensis* are cut into small pieces, brewed, and then mixed with *Citrus aurantiifolia* fruit juice	Taken two times a day	1
RBM08	Fresh *Curcuma longa* rhizomes are chopped and then boiled with fresh *Phyllanthus niruri* herbs	Taken two times a day	1
RBM09	Fresh *Kaempferia galanga* rhizomes are sautéed and then boiled with small pieces of *Tamarindus indica* fruit	Taken more than three times a day	1
RBM10	Fresh *Zingiber officinale* rhizomes are shredded, mixed with *Citrus aurantiifolia* fruit juice, and then brewed with hot water	Taken two times a day	1
RBM11	Fresh *Zingiber officinale* rhizomes are chopped and then boiled with *Cymbopogon citratus* herbs and *Cinnamomum burmanni* bark	Taken two times a day	3

*Tempurejo District, Jember Regency*			
RBJ01	Fresh rhizomes from *Zingiber officinale* and *Curcuma longa* are boiled or brewed together	Taken while coughing	1
RBJ02	*Citrus aurantiifolia* fruit juices are mixed with sweet soy sauce to taste	Taken while coughing	14
RBJ03	Fresh rhizomes from *Curcuma longa* and *Kaempferia galanga* are shredded and then squeezed	Taken while coughing	1
RBJ04	Fresh *Kaempferia galanga* rhizomes are shredded, then squeezed, and mixed with honey to taste	Taken while coughing	2
RBJ05	Fresh *Zingiber officinale* rhizomes are thinly sliced, then brewed, and mixed with honey to taste	Taken while coughing	1
RBJ06	Fresh *Zingiber officinale* var. *rubrum* rhizomes are boiled with *Cymbopogon citratus* herbs	Taken while coughing	1
RBJ07	Fresh *Peperomia pellucida* herbs are shredded with fresh *Kaempferia galanga* rhizomes and then squeezed	Taken while coughing	1
RBJ08	Fresh *Peperomia pellucida* herbs are boiled with fresh *Zingiber officinale* rhizomes	Taken while coughing	1
RBJ09	*Oryza sativa* seeds are soaked with water, drained, pounded with fresh *Kaempferia galanga* rhizomes, and then squeezed	Taken while coughing	1
RBJ10	Fresh rhizomes from *Kaempferia galanga*, *Curcuma zanthorrhiza*, and *Curcuma longa* are boiled with *Amomum compactum* fruit	Taken while coughing	1

**Table 3:** Diversity of medicinal plant species used in the treatment of coughs and colds in three selected regions of East Java Province.

**Family**	**Scientific name**	**Local name**	**Voucher number**	**Plant part used**	**Method of preparation**	**RA**	**Ailment (UR)**	**UV**	**IUCN status**
Apiaceae	*Centella asiatica* L.	Pegagan	EJ-2317	Leaves	Boiled	Oral	Colds (2)	0.018	LC
Asteraceae	*Elephantopus scaber* L.	Tapak liman	EJ-2314	Leaves	Boiled	Oral	Cough (1)	0.009	DD
Lamiaceae	*Mentha* x *piperita* L.	Mint	EJ-2323	Leaves	Crushed and then boiled	Oral	Colds (2)	0.018	DD
Lauraceae	*Cinnamomum burmanni* (Nees & T.Nees) Blume	Kayu manis	EJ-2319	Bark	Brewed or boiled	Oral	Cough (3); colds (1)	0.036	LC
Fabaceae	*Tamarindus indica* L.	Asam Jawa	EJ-2301	Fruit	Grated, boiled, or crushed and then boiled	Oral	Cough (4); colds (4)	0.063	LC
Liliaceae	*Allium sativum* L.	Bawang putih	EJ-2308	Bulb	Boiled or crushed and then brewed	Oral; inhalation	Cough (1); colds (2)	0.027	DD
Liliaceae	*Allium sativum* L. var solo garlic	Bawang putih jantan	EJ-2312	Bulb	Boiled	Oral	Cough (1)	0.009	DD
Oxalidaceae	*Averrhoa bilimbi* L.	Blimbing wuluh	EJ-2324	Flower; leaves	Brewed or boiled	Oral; inhalation	Cough (2)	0.018	LC
Piperaceae	*Peperomia pellucida* (L.) Kunth	Cekroh; Sirih Cina	EJ-2322	Herb	Boiled or grated and then squeezed	Oral	Cough (3)	0.027	DD
Piperaceae	*Piper nigrum* L.	Merica	EJ-2310	Seed	Boiled (if necessary, mixed with honey to taste)	Oral	Cough (3)	0.027	DD
Poaceae	*Cymbopogon citratus* (DC.) Stapf	Serai	EJ-2305	Leaves; herb	Boiled	Oral; inhalation	Cough (17); colds (13)	0.270	DD
Poaceae	*Oryza sativa* L.	Padi	EJ-2309	Seed	Boiled or crushed and then squeezed	Oral	Cough (3)	0.027	DD
Ranunculaceae	*Nigella sativa* L.	Jintan hitam; Habatussauda	EJ-2315	Seed	Crushed and then brewed or mixed with honey to taste	Oral	Cough (1); colds (2)	0.027	DD
Rubiaceae	*Morinda citrifolia* L.	Mengkudu	EJ-2325	Fruit	Boiled	Oral	Colds (1)	0.009	LC
Rutaceae	*Citrus aurantiifolia* (Christm.) Swingle	Jeruk nipis	EJ-2302	Fruit	Squeezed (if necessary, mixed with honey/sugar/sweet soy sauce), roasted, boiled, or squeezed and then brewed	Oral	Cough (26); colds (9)	0.315	DD
Solanaceae	*Capsicum frutescens* L.	Cabai rawit	EJ-2321	Leaves	Crushed and then squeezed	Oral	Colds (1)	0.009	LC
Verbenaceae	*Stachytarpheta jamaicensis* (L.) Vahl	Pecut kuda	EJ-2318	Leaves	Brewed	Oral	Cough (5)	0.045	LC
Zingiberaceae	*Amomum compactum* Sol. ex Maton	Kapulaga	EJ-2320	Fruit	Brewed, boiled, or crushed and then boiled	Oral	Cough (2); colds (5)	0.063	LC
Zingiberaceae	*Boesenbergia pandurata* (Roxb.) Schltr.	Temu kunci; Kunci	EJ-2316	Rhizome	Grated, boiled, or chopped and then brewed	Oral	Cough (1); colds (1)	0.018	DD
Zingiberaceae	*Curcuma longa* L.	Kunyit	EJ-2304	Rhizome	Brewed, boiled, grated, and then squeezed or chopped and then boiled/brewed	Oral	Cough (9); colds (11)	0.180	DD
Zingiberaceae	*Curcuma zanthorrhiza* Roxb.	Temulawak	EJ-2307	Rhizome	Boiled, grated, and then squeezed/brewed or chopped and then boiled	Oral	Cough (3); colds (3)	0.054	DD
Zingiberaceae	*Curcuma zedoaria* (Christm.) Roscoe	Kunyit putih	EJ-2313	Rhizome	Grated or boiled	Oral	Cough (1)	0.009	DD
Zingiberaceae	*Kaempferia galanga* L.	Kencur	EJ-2303	Rhizome	Eaten raw, brewed, boiled, grated, squeezed, crushed, and then squeezed or chopped and then boiled/brewed	Oral	Cough (27); colds (3)	0.270	DD
Zingiberaceae	*Zingiber officinale* Roscoe	Jahe	EJ-2306	Rhizome	Grated, brewed, boiled, or chopped and then brewed	Oral	Cough (22); colds (34)	0.486	DD
Zingiberaceae	*Zingiber officinale* Rosc. var. rubrum	Jahe merah	EJ-2311	Rhizome	Boiled	Oral	Cough (2)	0.018	DD

Abbreviation: DD = data deficient, LC = least concern, RA = route of administration, UR = use report, UV = use value.

## Data Availability

All data are available in this article.
