# Supplementary material for: Exploration of the Use of Traditional Herbs to Overcome Cough and Cold in Three Provinces of East Java Province
Source: ScientificWorldJournal. 2025 Apr 27;2025:1622754. doi: 10.1155/tswj/1622754 (PMC12050156; doi:10.1155/tswj/1622754)
Supplement: Supporting Information — Additional supporting information can be found online in the Supporting Information section. Research questionnaire. [file 1622754.f1.docx]

Research Questionnaire

"EXPLORATION OF THE USE OF TRADITIONAL HERBS TO OVERCOME COUGH AND COLD IN THREE PROVINCES OF EAST JAVA PROVINCE"

Data Collection Date

Data collection location:

Survey officer:

Dear Sir/Madam,

Please be willing to fill out this research questionnaire based on your knowledge and experience. Answers regarding your identity will be kept confidential and will only be used for research purposes. We sincerely appreciate your participation and assistance in filling out this questionnaire.

# How to fill out the questionnaire:

You can fill out the questionnaire sheet in the given column and if there is an option or answer choice, you can answer with the following conditions:

1. In the answer choice in the form of (a/b/c/d/e), you can only choose one answer by giving a cross (X) to the answer you think is appropriate.
2. In the answer choice in the form of a box (🞎), you can select more than one answer by checking (✓) the answer you think is appropriate. You can also check (✓) the “Other” answer choice and fill in your answer if the answer choice provided does not match.

# NOTE

This provision does not apply to the filling of the respondent's identity data. Filling in the respondent's identity data can only choose one of several options or answer choices by giving a check mark (✓) to the answer that you think is appropriate.

1. Respondent ID

| Full Name | : |  |  |
| --- | --- | --- | --- |
| Gender | : | ⬜Male | ⬜Female |
| Age | : |  |  |
| Religion | : | ⬜Buddhism  ⬜Hinduism  ⬜Islam | ⬜Catholic  ⬜Confucianism  ⬜Protestantism |
| Marital Status | : | ⬜Married | ⬜Unmarried |
| Educational backgrounds | : | ⬜No formal education  ⬜SD/MI/Equivalent  ⬜Junior High School/MTS/Equivalent  ⬜Senior High School/SMK/MA/Equivalent | ⬜Diploma I/II/III/IV  ⬜S1  ⬜S2  ⬜S3 |
| Main Employment Status | : | ⬜Students  ⬜Housewife  ⬜Farmer/Trader  ⬜Physician/Medical Expert  ⬜Religious Leaders  ⬜Civil servants | ⬜Private Sector Employee  ⬜Self-employed  ⬜TNI/Army  ⬜Medical Personnel  ⬜Retired  ⬜ Others |
| Monthly Income | : | ⬜< Rp 500,000  ⬜Rp500,000 –Rp1,000,000  ⬜Rp 1,000,000- Rp 2,000,000  ⬜Rp 2,000,000- Rp 3,000,000  ⬜> Rp 3.000.000 |  |
| Residential Address | : |  |  |
| Length of stay | : |  |  |
| Mobile No. | : |  |  |

1. HEALTH STATUS
2. Have you ever had COVID-19?
   1. Yes
   2. No
3. If “Yes”, are you experiencing symptoms caused by COVID-19 disease?
   1. Some are asymptomatic.
   2. Symptomatic (specify: …………………………………………………………………………)
4. Has anyone in your household been exposed to COVID-19?
   1. Yes
   2. No
5. If “Yes”, does the family experience symptoms caused by COVID-19?
   1. Some are asymptomatic.
   2. Symptomatic (specify: …………………………………………………………………………)
6. Do you have a history of certain diseases?
   1. Yes
   2. No
7. If “Yes”, what disease did you experience?

- Hypertension
- Diabetes
- my illness recurred
- Heart condition
- Lung disorders
- Renal impairment, nephritis
- Other (please specify): _______________

1. USE OF MEDICINAL PLANTS
2. Have you used traditional medicinal plants/herbs to treat a cold cough for at least the last 3 years from now?
   1. Yes
   2. No
3. Where did you get the knowledge of traditional medicinal plants/herbs to overcome the cold cough?
   1. Parents/Grandparents
   2. Brother/neighbor
   3. Traditional healers/other health workers
   4. Other (please specify)
4. Do you make your own traditional herbs that you use to overcome the cold cough?
   1. Yes
   2. No (specify product name: ………………………………………………………………….)
5. If “Yes”, what medicinal plants have you used to make the traditional potion? State the complete composition, if it consists of several medicinal plants.

| Disease | Response | Plant Parts | Total (g) |
| --- | --- | --- | --- |
| Cough |  |  |  |
|  |  |  |  |
|  |  |  |  |
|  |  |  |  |
|  |  |  |  |
| Runny nose |  |  |  |
|  |  |  |  |
|  |  |  |  |
|  |  |  |  |
|  |  |  |  |

1. Where did you get or obtain the medicinal plant material?

| Disease | Natural Habitat | Self-planted | Buy | Other |
| --- | --- | --- | --- | --- |
| Cough |  |  |  |  |
|  |  |  |  |  |
|  |  |  |  |  |
|  |  |  |  |  |
|  |  |  |  |  |
| Runny nose |  |  |  |  |
|  |  |  |  |  |
|  |  |  |  |  |
|  |  |  |  |  |
|  |  |  |  |  |

1. What is the condition of the medicinal plant material when used?

| Disease | Fresh | Dry | Other |
| --- | --- | --- | --- |
| Cough |  |  |  |
|  |  |  |  |
|  |  |  |  |
|  |  |  |  |
|  |  |  |  |
| Runny nose |  |  |  |
|  |  |  |  |
|  |  |  |  |
|  |  |  |  |
|  |  |  |  |

1. How do you process the traditional medicine/herb?

| Disease | Unprocessed/  Directly used | Braised / stewed | Shredded | Grounded | Other |
| --- | --- | --- | --- | --- | --- |
| Cough |  |  |  |  |  |
|  |  |  |  |  |  |
|  |  |  |  |  |  |
|  |  |  |  |  |  |
|  |  |  |  |  |  |
| Runny nose |  |  |  |  |  |
|  |  |  |  |  |  |
|  |  |  |  |  |  |
|  |  |  |  |  |  |
|  |  |  |  |  |  |

1. How to use the traditional medicinal plants/herbs?

| Disease | Drinking | | Brewed | Smeared | Exhaled | Other |
| --- | --- | --- | --- | --- | --- | --- |
|  | Before eating | After meals |  |  |  |  |
| Cough |  |  |  |  |  |  |
|  |  |  |  |  |  |  |
|  |  |  |  |  |  |  |
|  |  |  |  |  |  |  |
|  |  |  |  |  |  |  |
| Runny nose |  |  |  |  |  |  |
|  |  |  |  |  |  |  |
|  |  |  |  |  |  |  |
|  |  |  |  |  |  |  |
|  |  |  |  |  |  |  |

1. How many times are medicinal plants/traditional herbs given in a day?

| Disease | 1 x a day | 2 x a day | 3 x a day | Other |
| --- | --- | --- | --- | --- |
| Cough |  |  |  |  |
|  |  |  |  |  |
|  |  |  |  |  |
|  |  |  |  |  |
|  |  |  |  |  |
| Runny nose |  |  |  |  |
|  |  |  |  |  |
|  |  |  |  |  |
|  |  |  |  |  |
|  |  |  |  |  |

1. How long is the treatment with traditional medicinal plants/herbs done?

| Disease | Until healed | No more than 1 weeks old | No more than 2 weeks | Other |
| --- | --- | --- | --- | --- |
| Cough |  |  |  |  |
|  |  |  |  |  |
|  |  |  |  |  |
|  |  |  |  |  |
|  |  |  |  |  |
| Runny nose |  |  |  |  |
|  |  |  |  |  |
|  |  |  |  |  |
|  |  |  |  |  |
|  |  |  |  |  |

1. What are the benefits felt after using the traditional medicinal plants/herbs? What is lina doing

| Disease | Decreased complaints | Easy to sleep | Increased appetite | Other |
| --- | --- | --- | --- | --- |
| Cough |  |  |  |  |
|  |  |  |  |  |
|  |  |  |  |  |
|  |  |  |  |  |
|  |  |  |  |  |
| Runny nose |  |  |  |  |
|  |  |  |  |  |
|  |  |  |  |  |
|  |  |  |  |  |
|  |  |  |  |  |

1. Are there any unwanted effects or side effects after using the traditional medicinal plants/herbs? What is lina doing

| Disease | None | Nausea and vomiting | Body feels floating and light | Other |
| --- | --- | --- | --- | --- |
| Cough |  |  |  |  |
|  |  |  |  |  |
|  |  |  |  |  |
|  |  |  |  |  |
|  |  |  |  |  |
| Runny nose |  |  |  |  |
|  |  |  |  |  |
|  |  |  |  |  |
|  |  |  |  |  |
|  |  |  |  |  |
